# Inverse association of colorectal cancer prevalence to serum levels of perfluorooctane sulfonate (PFOS) and perfluorooctanoate (PFOA) in a large Appalachian population

**DOI:** 10.1186/1471-2407-14-45

**Published:** 2014-01-27

**Authors:** Kim E Innes, Jeffrey H Wimsatt, Stephanie Frisbee, Alan M Ducatman

**Affiliations:** 1 Department of Epidemiology, West Virginia University School of Public Health, PO Box 9190, Morgantown, WV 26506-9190, USA; 2 University of Virginia Health System, Charlottesville, Virginia 22908-0782, USA; 3 Department of Medicine, West Virginia University School of Medicine, Morgantown, WV 26506-9190, USA; 4 Department of Health Policy, Management and Leadership, West Virginia University School of Public Health, PO Box 9190, Morgantown, WV 26506-9190, USA; 5 Department of Environmental and Occupational Health, West Virginia University School of Public Health, Morgantown, WV 26506-9190, USA; 6 Department of Medicine and Cancer Center, West Virginia University School of Medicine, Morgantown, WV 26506-9190, USA

**Keywords:** Colorectal cancer epidemiology, Colon cancer, Perfluorooctanoate (PFOA), Perfluorooctane sulfonate (PFOS), Perfluorocarbons, Perfluoroalkyl acids, Peroxisome proliferator-activated receptors (PPARs), Gender, Body mass index, Inflammation

## Abstract

**Background:**

Perfluorooctanoate (PFOA) and perfluorooctane sulfonate (PFOS) are persistent environmental contaminants that affect metabolic regulation, inflammation, and other factors implicated in the development and progression of colorectal cancer (CRC). However, the link between these compounds and CRC remains unknown. In this cross-sectional study, we investigated the association of CRC diagnosis to PFOA and PFOS blood levels in a large Appalachian population.

**Methods:**

Participants were 47,359 adults ≥ 21 years of age and residing in six PFOA-contaminated water districts in the mid-Ohio Valley (N = 47,151 cancer-free adults, 208 cases of primary CRC). All participants completed a comprehensive health survey between 2005 and 2006; serum levels of PFOA, PFOS, and a range of other blood markers were also measured. Medical history was assessed via self report and cancer diagnosis confirmed via chart review.

**Results:**

CRC showed a strong inverse, dose–response association with PFOS serum levels (odds ratio (OR) adjusted for potential confounders = 0.2, 95% confidence interval (CI) 0.2,0.3) for highest vs. lowest quartile of PFOS, *P*-trend < 0.00001) and a significant, but more modest inverse association with PFOA (adjusted OR = 0.6 (CI 0.4, 0.9) for highest vs. lowest quartile, *P*-trend = 0.001). These inverse associations were stronger in those diagnosed within the previous 6 years and resident in the same water district for a minimum of 10–15 years preceding assessment. The relationship between PFOA and CRC was also more pronounced in men and leaner adults, and showed a stronger linear trend at lower exposure levels.

**Conclusions:**

In this large cross-sectional study, we found a strong, inverse association between PFOS and likelihood of CRC diagnosis and a significant, although more modest inverse association between PFOA and CRC. If confirmed in prospective investigations, these findings may aid in identifying new strategies for CRC prevention and treatment and inform future studies regarding mechanisms underlying CRC pathogenesis.

## Background

While incidence and mortality rates of colorectal cancer (CRC) have declined during the past decade, CRC remains the third most common cancer in both men and women and the third leading cause of cancer-related mortality in the United States [1,2]. Major risk factors for CRC include age, a family or personal history of CRC, colorectal polyps, chronic inflammatory bowel disease, and inherited genetic alterations, such as familial adenomatous polyposis or hereditary nonpolyposis CRC (Lynch syndrome) [2,3]. CRC rates are higher in men and in African American populations [2,3]. Certain lifestyle-related factors also increase risk for CRC, including physical inactivity, obesity, smoking, and a diet high in red and processed meats [2,3]. Recent cohort studies suggest constipation may also increase CRC risk [4,5]. Conversely, a growing body of evidence suggests that use of aspirin and other anti-inflammatory medications [6-9] and certain dietary supplements (e.g., calcium) may be protective against CRC [2]. In addition, the role of peroxisome proliferator-activated receptor-*γ* (PPAR-*γ*) in adipocyte differentiation, the antiproliferative and/or differentiating effects of PPAR-α and PPAR-γ ligands in human colon and other tumor cell lines, and the anticancer effects of both PPAR isotypes in animal models of CRC support a chemoprotective role for these nuclear hormone receptors [10,11].

Certain environmental contaminants have also been linked to increased risk for incident CRC, including drinking water nitrate [12] and chloroform levels [13]. However, the link between CRC and other widespread contaminants, including perfluorocarbon compounds (PFC’s), remains unclear [14,15]. Given the documented protective role of non-steroidal anti-inflammatory medications [6], and the growing number of studies supporting a role for PPARs in CRC prevention and treatment [10,11], it is possible that certain perfluoroalkyl acids (PFAAs), including the widespread pollutants, perfluoroctanoic acid (PFOA) and perfluorooctanesulfonic acid (PFOS), may also be associated with reduced CRC risk. These compounds are potent PPAR ligands, and have demonstrated anti-inflammatory effects in vitro [16] and in animal studies [17] that are thought to operate via both PPAR-dependent and –independent pathways [18]. To date, only two studies in the same cohort of fluorochemical and film plant employees have assessed the association of PFAAs or any other PFCs to CRC: a survey study of 1400 workers [14] and an overlapping analysis of health claims data from 1301 employees [15]. While neither study documented significant associations between PFOS and CRC, conclusions were limited by very small numbers (N = 12 confirmed CRC cases), reliance on self-report or claims data, and lack of information on PFOS blood levels or potential confounders.

In this study, we investigated the association of prevalent colorectal cancer to PFOA and PFOS in a large Appalachian population who were exposed to elevated levels of PFOA through contaminated drinking water.

## Methods

### Study population

The population for this study were adult participants in the C8 Health Study Project [19,20], a study which resulted from the settlement of a class-action lawsuit related to the widespread PFOA contamination of drinking water by a large production facility located in Washington, West Virginia. PFOA production began in the 1950’s, with water contamination first observed in the 1980’s [21]. From August 2005 to August 2006, baseline data were gathered on 69,030 individuals living or working in six PFOA-contaminated water districts in Ohio and West Virginia, including those exposed to contaminated private-well drinking water. The first water filtration and other abatement procedures were instituted in 2007 [21]. Project details, including those regarding consent, enrollment, data collection and reporting, have been published [20] and are described online (http://publichealth.hsc.wvu.edu/c8/). In 2008, investigators in the WVU Department of Community Medicine (now the WVU School of Public Health) were granted formal access to the raw deidentified project data by Brookmar, the organization responsible for conducting the C8 health project (see http://www.hpcbd.com/C8%20Brookmar%20Health%20Project.html), and obtained approval from the West Virginia University Institutional Review Board to allow cleaning, coding, analysis and publication of these data.

Estimated participation rate in the C8 Health Project among adult residents of the affected water districts was 81% [19]. For this study, eligible participants included all adults aged ≥21 years of age at the time of baseline assessment, who had not received a diagnosis of cancer other than colon or rectal cancer, and who had complete data on all covariates of interest. Cases included those with a medical-record confirmed diagnosis of colon and/or rectal cancer. Details of sample selection are given in below.

### Outcome and exposure measurements

Participants in the C8 Health Project completed a comprehensive health survey and blood tests to determine clinical biomarkers and serum levels of the primary exposures of interest, PFOA, PFOS, in addition to eight other perfluorocarbon compounds (see below) [20]. These latter compounds included PFPeA (C5), PFHxA (C6), PFHS (C6s), PFHpA (C7), PFNA (C9), PFDA (C10), PFUnA (C11), and PFDoA (C12). Medical history, including physician diagnosis of medical conditions, was assessed via self-report questionnaires. Diagnosis of cancer and cancer type, as well as diagnoses of certain other clinical disorders, including diabetes and cardiovascular disease, were further verified via chart review. Demographic, lifestyle, and anthropometric characteristics were also determined via self-report; demographic data and health survey completion were verified by trained project staff.

### Laboratory methods: ascertainment of PFOA and PFOS

Blood processing, assay methods, and quality-assurance measures are described in detail elsewhere [19,20,22]. All assay methods, assay validations, and lab procedures were in strict adherence to Food and Drug Administration (FDA) approved standards [23]. In brief, blood samples were collected from each participant, serum was separated from red cells, and the samples were immediately refrigerated at collection and transported on dry ice to the laboratory for analysis. PFAA assays used a protein precipitation extraction method with reverse-phase high-performance liquid chromatography/tandem mass spectrometry. Detection was performed using a triple quadrupole mass spectrometer in selected reaction monitoring mode, monitoring for the M/Z transitions of 10 individual perfluorocarbon compounds and an internal ^13^C-PFC standard corresponding to each target compound analyzed. All laboratory analyses were performed using FDA bioanalytical method validation procedures [23]. Results of all assays were transferred automatically into the project’s Windows-based information system to prevent transcription errors. Of the PFCs tested, four (perfluorohexane sulfonic acid (PFHS or C6s), PFOA (C8), PFOS (C8s), perfluorononanoic acid (PFNA or C9) were detectable in almost all (> 97%) samples; for these compounds, test results reported as less than the limit of detection (LOD) were substituted with 0.25 ng/mL (50% of the lower LOD of 0.5 ng/mL). Three PFCs (Perfluorohexanoic acid (PFHxA or C6), perfluoroheptanoic acid (PFHpA or C7), perfluorodecanoic acid (PFDA or C10)) were detectable in approximately 50% of the samples; for these PFCs, no substitutions for values were included in the analyses [20].

### Statistical analysis

Data were analyzed using SPSS version 20. We used logistic regression analysis to assess the independent associations of PFOA and PFOS serum levels and other factors to CRC diagnosis, to evaluate the influence of potential confounders, and to examine potential effect modifiers. Linear trends were evaluated using polynomial contrasts. Potential differences between participants with and without missing data were assessed using the Students T test or Mann–Whitney U Test (for continuous or ordinal variables) and the chi square test (for categorical variables). The primary explanatory variables of interest, PFOA and PFOS, were analyzed as both continuous and categorical variables (study population quartiles and ventiles, with the lowest percentile group used as referent category). All p-values shown are 2-sided.

Factors on which adequate data were available and which have been previously linked to CRC and/or the two PFAAs of interest were selected a priori as covariates. Associations of PFAAs to CRC were initially adjusted for age, a factor strongly associated with both PFOA and PFOS levels and CRC. Unless stated otherwise, all other multivariable models were adjusted for age, sex, race/ethnicity, marital status, socioeconomic status (SES, including years of education, average family income, and employment status/disability), participation in a regular exercise program (yes/no), vegetarian diet (yes/no), smoking (never, former, current), current alcohol consumption (yes/no), menopausal status and use of hormone replacement therapy (women), body mass index (BMI, calculated as kg/m^2^), medical comorbidity (reported physician diagnosis of other medical conditions, including heart, kidney, liver, thyroid, immune, and connective tissue disease, stroke, hypertension, dyslipidemia, diabetes, chronic obstructive pulmonary disease, or asthma), and current treatment for hypertension or hyperlipidemia. Additional analyses adjusted for arthridides (self-reported physician diagnosis of rheumatoid arthritis, osteoarthritis, or fibromyalgia); gastrointestinal symptoms that could be associated with reduced absorption (abdominal pain, nausea, diarrhea, indigestion, and bloody stools); anemia (hemoglobin < 12 g/dL in women and <13.5 g/dL in men); and serum levels of folate (ng/mL), cholesterol (mg/dL), C-reactive protein (mg/L), uric acid (mg/dL), estradiol (pg/mL), and other PFAAs measured in the C8 Health Project.

To evaluate potential modifying effects of gender, BMI (<30, ≥30), treatment method (chemotherapy/radiation vs. no chemotherapy/radiation), and years since diagnosis (before 2000 vs. 2000 or later) on the association of PFAA levels (in quartiles) to history of CRC, we conducted multivariable analyses stratified by each potential effect modifier. We tested the strength of each interaction by including the corresponding multiplicative interaction term in the main adjusted statistical model and evaluating the coefficient using the Wald test. In addition, to assess the robustness of the observed associations, we conducted additional sensitivity analyses to evaluate the relation of CRC diagnosis to PFOA and PFOS at differing levels of exposure and to assess the relation of recently diagnosed CRC to these PFAAs in participants who were long term residents of the affected water districts. To help assess whether observed associations of PFOA and/or PFOS could be due reverse causality (e.g., reduced absorption), we conducted additional analyses adjusting for anemia and specific GI symptoms (frequent diarrhea, bloody stool, abdominal pain, nausea, and/or constipation) as well as analyses excluding CRC cases who were currently under treatment or had received chemotherapy. We also assessed the association of CRC prevalence to other gut-absorbed compounds, including folate and other PFCs for which adequate data were available (including C6, C6s, C7, C9, and C10).

## Results

**Figure 1:**
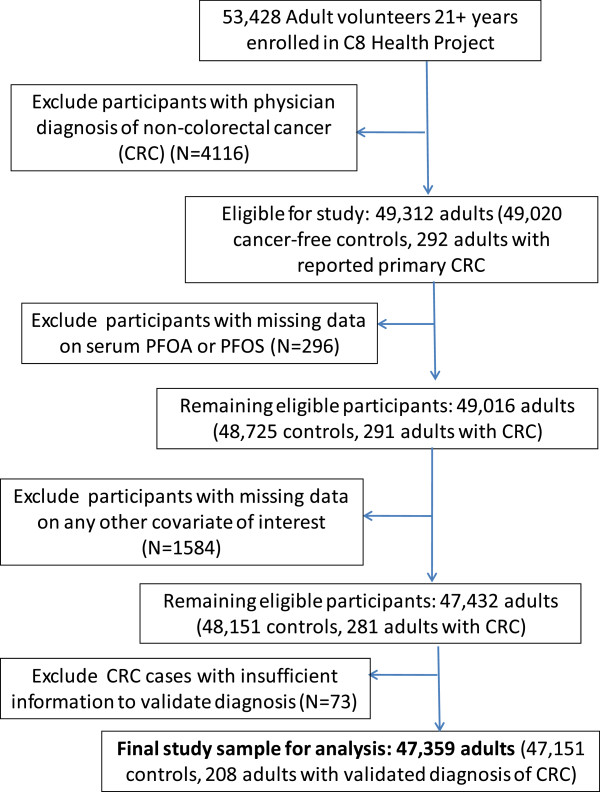
Study flow diagram.

The study flow diagram is given in Figure 1. Participants who had received a diagnosis of cancer other than primary colon or rectal cancer (N = 4116) were excluded from the analytic sample, leaving a total of 49,312 eligible adults. Exclusion of participants with missing data on PFOA and PFOS (N = 296, 0.6%) and other covariates of interest (N = 1584, 3.2%) and of colorectal cancer cases that could not be validated due to missing information (N = 73) yielded a final study sample of 47,359, including 47,151 cancer-free controls and 208 adults with a medical record-confirmed diagnosis of primary colon or rectal cancer. Among validated CRC cases with information on diagnosis date (N = 193), 99 were diagnosed prior to 2000, and 94 received a diagnosis in 2000 or later (median 1999, range 1966 to 2006). Compared to participants included in the analyses, those with missing data on any covariate were more likely to be female and postmenopausal, to be older, less educated and heavier, and to report lower income and higher prevalence of comorbid medical conditions (P < 0.01).

The distribution of study population characteristics by CRC diagnosis is given in Table 1. Participants ranged in age from 21 to 105 years of age (mean (SD) = 45.71 (15.0) years), and were predominantly (97%) non-Hispanic white. Fifty-two percent were female, 38% reported an annual household income of less than $30,000, and 53% had received only 12 years of schooling or less. Sixty-three percent were employed, and 7% were disabled. Over 70% of the adults in this Appalachian population were overweight (BMI 25 or greater), and over 35% were obese (BMI ≥30).

**Table 1:** **Characteristics of adults** ≥ **21 years of age from 6 PFOA**-**contaminated water districts in the Ohio Valley**, **stratified by diagnosis of colorectal cancer** (**N**=**208 colorectal cancer cases**, **47**,**151 cancer**-**free controls**)

	***Diagnosis of colorectal cancer***				***Adjusted OR*** ** ( *95% CI *)	***P***-***value*** *
	***No* ( *N *** **= *47 *, *151 *)**		***Yes* ( *N *** **= *208 *)**			
	***N***	**%**	***N***	**%**		
***Demographic characteristics***						
Age (Years)						<0.00001
*Mean* (*SD*)	45.49 (14.97)		67.92 (10.80)			
Per year increment					1.10 (1.07,1.11)	<0.00001
Gender						0.05
Female	24292	51.52%	94	45.19%	1.00 (Referent)	
Male	22859	48.48%	114	54.81%	1.40 (1.00,1.57)	
Ethnicity						0.12
White	45835	97.33%	207	99.52%	1.00 (Referent)	
Minority	1256	2.67%	1	0.48%	0.21 (0.03,1.51)	
Marital status						0.53
Married/co-habiting	34170	72.47%	157	75.48%	1.00 (Referent)	
Single	6105	12.95%	6	2.88%	0.81 (0.35,1.88)	
Divorced/separated	5145	10.91%	14	6.73%	0.83 (0.47,1.47)	
Widowed	1731	3.67%	31	14.90%	0.72 (0.46,1.15)	
Years of education						.
<12 years	5102	10.82%	50	24.04%	1.00 (Referent)	0.85
High school/GED	19674	41.73%	91	43.75%	0.89 (0.60,1.31)	
Some college	15777	33.46%	51	24.52%	0.97 (0.60,1.55)	
4+ years college	6598	13.99%	16	7.69%	0.79 (0.40,1.54)	
Current employment status						0.29
Employed	29704	63.53%	42	20.19%	1.00 (Referent)	
Homemaker	5411	11.57%	30	14.42%	1.53 (0.88,2.68)	
Retired	5679	12.15%	115	55.29%	1.75 (1.10,2.77)	
Unemployed/Laid off	1992	4.26%	3	1.44%	1.83 (0.56,5.97)	
Student	833	1.78%	0	0.00%	0.00 (0.00,0.00)	
Disabled	3132	6.70%	17	8.17%	1.94 (1.06,3.57)	
Other	4	0.01%	1	0.48%	1.20 (0.16,9.09)	
Average household income						0.49
≥ $30, 000	25269	53.59%	88	42.31%	1.00 (Referent)	
< $30, 000	17635	37.40%	103	49.52%	1.05 (0.78,1.41)	
Don’t know	4247	9.01%	17	8.17%	1.07 (0.70,1.63)	
Ever consumed alcohol						0.46
No	23348	49.52%	91	43.75%	1.00 (Referent)	
Yes	23803	50.48%	117	56.25%	1.08 (0.83,1.47)	
Smoking status						0.06
Never	22305	47.31%	101	48.56%	1.00 (Referent)	
Former	12298	26.08%	91	43.75%	1.04 (0.76,1.43)	
Current	12548	26.61%	16	7.69%	0.51 (0.29,0.89)	
Regular exercise program						0.59
No	32512	68.95%	139	66.83%	1.00 (Referent)	
Yes	14639	31.05%	69	33.17%	0.96 (0.73,1.26)	
Vegetarian diet						0.53
No	*46802*	99.26%	205	98.56%	1.00 (Referent)	
Yes	*349*	0.74%	3	1.44%	1.46 (0.53,4.68)	
***Anthropometrics and medical history***						
BMI (kg/m2)						0.47
< 24.99	13698	29.10%	51	24.52%	1.00 (Referent)	
25-29.99	16600	35.27%	91	43.75%	1.32 (0.93,1.89)	
30-34.99	9912	21.06%	42	20.19%	1.19 (0.78,1.83)	
≥ 35	6859	14.57%	24	11.54%	1.33 (0.79,2.25)	
Per unit increment BMI					1.02 (0.99,1.04)	0.21
*Mean* (*SD*)		*28.80* (*6.40*)		*28.35* (*5.82*)		
Comorbid condition(s)ŧ						0.005
No	30299	64.26%	71	34.13%	1.00 (Referent)	
Yes	16852	35.74%	137	65.87%	1.56 (1.14,2.13)	
On lipid-lowering medication						0.03
No	31949	67.76%	132	63.46%	1.00 (Referent)	
Yes	15202	32.24%	76	36.54%	0.72 (0.52,0.97)	
On anti-hypertensive medication						0.29
No	34990	74.21%	103	49.52%	1.00 (Referent)	
Yes	12161	25.79%	105	50.48%	0.79 (0.58,1.07)	
*Reproductive history* (*women*, *N*=*24292 controls*, *94 cases*)						
Postmenopause						0.003
No	14361	59.12%	4	4.26%	1.00 (Referent)	
Yes	8935	36.78%	88	93.62%	7.64 (2.35,24.79)	
Don’t know	996	4.10%	2	2.13%	3.93 (0.70,22.06)	
History of hormone replacement therapy						0.08
No	16973	69.87%	57	60.64%	1.00 (Referent)	
Yes	7319	30.13%	37	39.36%	0.66 (0.42,1.05)	

*All p-values are 2-sided **Adjusted for other factors in table.

ŧReported physician diagnosis of other medical conditions, including heart, kidney, liver, thyroid, immune, and connective tissue disease, stroke, hypertension, dyslipidemia, diabetes, chronic obstructive pulmonary disease, or asthma.

*Abbreviations*: *BMI* Body mass index, *CI* Confidence intervals, *PFOA* perfluorooctanoate, *OR* Odds ratio, *SD* Standard deviation.

Of the 47,359 eligible participants with no missing data, 208 were diagnosed with CRC. After adjustment for all other factors in the table, CRC retained significant, positive associations with age (p < 0.00001) and male gender (p < 0.05), and with postmenopausal status in women (P <0.003). Participants who were disabled or had been diagnosed with at least one chronic medical condition were also significantly more likely to have been diagnosed with CRC (P < 0.02). Conversely, those who were on lipid-lowering medications were less likely to have received a diagnosis of CRC (P <0.02).

Serum values of PFOA were elevated in this population, averaging 86.6 (255.1) ng/mL (median = 27.9, range <0.5-22,412 ng/mL), compared to a geometric mean of 3.7-4.2 ng/mL across adult age ranges in the 2003–2004 NHANES population [24]. In contrast, PFOS serum levels in our study sample were similar to those in the general U.S. population [24], ranging from <0.5 to 759.2 ng/mL and averaging 23.4 ± 16.3 ng/mL (median = 20.2 ng/mL). Likewise, serum levels of other PFAA’s for which adequate data were available were comparable to general background levels in the U.S [24,25].

Table 2 details the associations between CRC diagnosis and serum levels of PFOA and PFOS. PFOS showed a strong, inverse association to diagnosis of CRC in both the minimally adjusted analysis and the full models. Those testing in the highest PFOS quartile were 80% less likely to have been diagnosed with CRC than those in the lowest quartile (odds ratio (OR) = 0.2, 95% confidence interval (CI) 0.1, 0.3, P for trend < 0.00001) after adjustment for age and BMI. Further adjustment for sociodemographic characteristics, menopausal status and hormone replacement therapy (HRT) use, lifestyle factors, and comorbidity did not materially alter these risk estimates (OR’s = 0.35 (CI 0.2, 0.5), 0.3 (CI 0.2, 0.5), and 0.2 (CI 0.2, 0.3) for the second, third, and highest quartile of PFOS, respectively; P for trend < 0.00001), nor did additional adjustment for gastrointestinal (GI) symptoms and for serum levels of folate, estradiol, cholesterol, uric acid, and C-reactive protein. The association of CRC to serum PFOS analyzed as a continuous variable showed a similar pattern, with the strength of the linear relationship remaining constant after adjustment for other demographic, lifestyle, and health-related factors (P < 0.00001, Table 2).

**Table 2:** **Association of serum perfluorooctanoate** (**PFOA**) **and perfluorooctane sulfonic acid** (**PFOS**) **levels to colorectal cancer diagnosis** (**N**=**208 colorectal cancer cases**, **47**,**151 cancer free controls**) **in adults aged 21 and older**

	**CRC cases (N)**	**Controls (N)**	**Adjusted for age**				Adjusted for age, race, gender, SES, marital status, lifestyle factors*, BMI, menopausal status, and comorbidityŧ				Also adjusted for metabolic/physiologic profile** and gastrointestinal symptoms¥			
			**Odds ratio**	**95% CI**		**P**	**Odds ratio**	**95% CI**		**P**	**Odds ratio**	**95% CI**		**P**
				**Lower**	**Upper**			**Lower**	**Upper**			**Lower**	**Upper**	
*PFOS quartiles*														
First (0.25-13.5 ng/mL)	79	11657	1.00	Referent			1.00	Referent			1.00	Referent		
Second (13.6-20.1 ng/mL)	39	11788	0.39	0.26	0.57	<0.00001	0.35	0.24	0.53	<0.00001	0.38	0.25	0.59	<0.00001
Third (20.2-29.1 ng/mL)	42	11838	0.33	0.23	0.48	<0.00001	0.30	0.20	0.45	<0.00001	0.27	0.17	0.42	<0.00001
Fourth (≥ 29.2 ng/mL)	48	11868	0.27	0.19	0.39	<0.00001	0.23	0.15	0.34	<0.00001	0.24	0.16	0.37	<0.00001
* Test for trend*						<0.00001				<0.00001				<0.00001
* Per unit increase PFOS* (*ng*/*mL*)			0.97	0.96	0.98	<0.00001	0.96	0.95	0.97	<0.00001	0.96	0.95	0.97	<0.00001
*PFOA quartiles*														
First (0.25-13.4 ng/mL)	58	11588	1.00	Referent			1.00	Referent			1.00	Referent		
Second (13.5-27.8 ng/mL)	36	11988	0.50	0.33	0.77	0.001	0.47	0.31	0.74	0.001	0.48	0.31	0.75	0.001
Third (27.9-71.2 ng/mL)	49	11796	0.53	0.36	0.78	0.001	0.49	0.33	0.74	0.001	0.51	0.34	0.77	0.001
Fourth (≥ 71.3 ng/mL)	65	11779	0.64	0.45	0.92	0.02	0.61	0.42	0.89	0.01	0.64	0.44	0.94	0.02
* Test for trend*						0.002				0.001				0.002
*Per unit increase PFOA* (*ng*/*mL*)			1.00	1.00	1.00	0.42	1.00	1.00	1.00	0.35	1.00	1.00	1.00	0.46

*Abbreviations*: *BMI* body mass index (kg/m2), *CI* Confidence interval, *CRP* C-reactive protein, *HRT* hormone replacement therapy, *SES* socioeconomic status.

*Lifestyle factors include smoking (never, former, current); current alcohol consumption (yes/no); vegetarian diet (yes/no); exercise (regular exercise program (yes/no)); SES includes years of education, annual household income, and employment status/disability.

ŧComorbidity includes physician diagnosis of comorbid conditions (heart, kidney, liver, immune, connective tissue, and thyroid disease, stroke, hypertension, dyslipidemia, diabetes, chronic obstructive pulmonary disease, or asthma) and current treatment for hypertension or hyperlipidemia.

**Serum lipid profiles, C-reactive protein, estradiol, and uric acid.

¥Diarrhea, abdominal pain, nausea, bloating, blood stools, and indigestion.

PFOA also demonstrated a significant, inverse, although more modest relationship to CRC (Table 2). Participants in the highest PFOA quartile showed an approximately 40% reduced likelihood of CRC diagnosis (age-adjusted OR = 0.6, CI 0.4, 0.9, P for trend = 0.002), although the linear trend was weak and non-significant when PFOA was analyzed as a continuous variable (p = 0.42). Additional adjustment for other demographic, socioeconomic, lifestyle, and health-related factors did not appreciably alter the strength or magnitude of this inverse association.

Further adjustment for other PFAAs, for anemia, or for diagnosed osteoarthritis, rheumatoid arthritis, and fibromyalgia or did not alter these associations, nor did exclusion of those with low hemoglobin levels (N = 2391, including 41 CRC cases).

As detailed in Tables 3, 4 and 5, the protective association of PFOA was more pronounced in men than in women (age-adjusted OR’s, respectively for the highest vs. the lowest quartile = 0.5 (CI 0.3, 0.9) vs. 0.8 (CI 0.4, 1.3), p for interaction < 0.05) and tended to be stronger in leaner (BMI < 30) than in obese adults (BMI 30+) (fully adjusted OR’s, respectively, for highest vs. lowest quartile = 0.5 (CI 0.3, 0.9) vs. 0.9 (CI 0.5, 1.8), p for interaction < 0.09). Similarly, the inverse relation of PFOA to prevalent CRC was significantly stronger in those diagnosed within the previous 6 years (2000 or later) relative to those diagnosed earlier (adjusted OR’s, respectively for the highest vs. the lowest quartile = 0.4 (CI 0.3, 0.7) vs. 0.9 (CI 0.5, 1.6)). The association of PFOS to likelihood of CRC was also more pronounced in those diagnosed more recently (adjusted OR’s, respectively, for highest vs. lowest quartile = 0.1 (CI 0.1, 0.2) vs. 0.4 (CI 0.3, 0.7)) (Table 5). We did not find evidence for a modifying effect of gender or BMI on the relation of CRC to PFOS, or of age or CRC treatment method on the association of CRC to either PFOA or PFOS.

**Table 3:** **Association of serum PFOS and PFOA levels to CRC diagnosis by gender in adults** ≥ **21 years of age**

	**Men only (N=114 cases)**						**Women only (N=94 cases)**						**P for interaction**
	**Cases**	**Controls**	**Odds ratio**	**95% CI**		**P**	**Cases**	**Controls**	**Odds ratio**	**95% CI**		**P**	
				**Lower**	**Upper**					**Lower**	**Upper**		
***PFOS quartiles***													
First (reference)	40	3338	1.00				39	8319	1.00				0.30
Second	22	5232	0.34	0.20	0.59	0.00001	17	6556	0.38	0.21	0.67	0.001	
Third	25	6785	0.28	0.17	0.47	<0.00001	17	5053	0.32	0.18	0.57	0.0001	
Fourth	27	7504	0.20	0.12	0.34	<0.00001	21	4364	0.31	0.18	0.53	0.00002	
*Test for trend*						<0.00001						<0.00001	
***PFOA quartiles***													
First (reference)	33	4016	1.00				25	7572	1.00				0.04
Second	16	5909	0.35	0.19	0.64	0.001	20	6079	0.70	0.38	1.26	0.23	
Third	29	6203	0.49	0.29	0.81	0.006	20	5593	0.56	0.31	1.02	0.06	
Fourth	36	6731	0.53	0.32	0.86	0.01	29	5048	0.76	0.44	1.32	0.33	
*Test for trend*						0.002						0.32	

**Table 4:** **Association of serum PFOS and PFOA levels to CRC cancer diagnosis in adults** ≥ **21 years of age**, **stratifed by BMI**

	**Obese (BMI ≥ 30, N = 66 cases)**						**Non-obese (BMI < 30, N = 142 cases)**						**P for interaction**
	**Cases**	**Controls**	**Odds ratio**	**95% CI**		**P**	**Cases**	**Controls**	**Odds ratio**	**95% CI**		**P**	
				**Lower**	**Upper**					**Lower**	**Upper**		
***PFOS quartiles***													
First (reference)	27	4273	1.00				52	7384	1.00				0.65
Second	10	4223	0.26	0.13	0.55	0.0004	29	7565	0.42	0.26	0.67	0.0002	
Third	14	4205	0.30	0.16	0.58	0.0004	28	7633	0.30	0.19	0.49	<0.00001	
Fourth	15	4086	0.23	0.12	0.44	<0.00001	33	7782	0.25	0.16	0.39	<0.00001	
*Test for trend*						<0.00001						<0.00001	
***PFOA quartiles***													
First (reference)	16	4395	1.00				42	7193	1.00				0.09
Second	12	4452	0.62	0.29	1.32	0.22	24	7536	0.44	0.26	0.73	0.002	
Third	16	4100	0.69	0.34	1.39	0.30	33	7696	0.45	0.28	0.72	0.001	
Fourth	22	3840	0.87	0.45	1.67	0.67	43	7939	0.52	0.34	0.80	0.003	
*Test for trend*						0.57						0.001	

**Table 5:** Association of serum PFOA and PFOS levels to CRC diagnosis in adults 21+years, stratified by year of diagnosis

	Diagnosis in 2000 or later (N=94 cases)**						Diagnosis before 2000 (N=99 cases)**						**P for interaction**
	**Cases**	**Controls**	**Odds ratio**	**95% CI**		**P**	**Cases**	**Controls**	**Odds ratio**	**95% CI**		**P**	
				**Lower**	**Upper**					**Lower**	**Upper**		
***PFOS quartiles***													
First (reference)	50	11657	1.00				27	11657	1.00				0.04
Second	15	11788	0.22	0.12	0.39	<0.00001	20	11788	0.56	0.31	1.00	0.05	
Third	16	11838	0.18	0.10	0.31	<0.00001	23	11838	0.49	0.28	0.87	0.015	
Fourth	13	11868	0.10	0.05	0.19	<0.00001	29	11868	0.44	0.25	0.74	0.002	
*Test for trend*						<0.00001						0.015	
***PFOA quartiles***													0.02
First (reference)	35	11588	1.00				21	11588	1.00				
Second	14	11988	0.32	0.17	0.59	0.0003	18	11988	0.68	0.36	1.27	0.23	
Third	19	11796	0.34	0.19	0.59	0.0002	25	11796	0.71	0.39	1.28	0.26	
Fourth	26	11779	0.41	0.25	0.68	0.0007	35	11779	0.91	0.53	1.58	0.74	
*Test for trend*						0.00004						0.50	

*Table 3-5. Abbreviations*: *BMI* body mass index (kg/m2), *CI* Confidence interval, *CRC* colorectal cancer, ***PFOA*** perfluorooctanoate, ***PFOS*** perfluorooctanesulfonic acid.

* All analyses adjusted for sociodemographic and lifestyle factors, BMI, menopausal status, and comorbidity. Sociodemographics include age, race/ethnicity, gender, education, income, marital status, and employment status; lifestyle factors include smoking status; current alcohol consumption; regular exercise program; comorbidity includes physician diagnosis of comorbid conditions (heart, kidney, liver, immune, connective tissue, and thyroid disease, stroke, hypertension, dyslipidemia, diabetes, chronic obstructive pulmonary disease, or asthma) and current treatment for hypertension or hyperlipidemia.

**Information on year of diagnosis missing for 15 CRC cases.

Similarly, as illustrated in Table 6, restricting the analysis to those who had lived at the same residence since 1990–1995 or before and to CRC cases diagnosed in 2000 or later strengthened the inverse associations with both PFAAs. Adjusted OR’s for highest vs. lowest quartile were 0.1 (CI 0.1, 0.2) for PFOS and 0.4 (CI 0.2, 0.5) for PFOA. Further restricting CRC cases to those diagnosed in 2005–6 (N = 15 CRC cases) yielded similar findings (adjusted OR’s for highest vs. lowest quartile = 0.1 (CI 0.0, 0.5) for PFOS and 0.4 (CI 0.1, 1.4) for PFOA).

**Table 6:** **Association of serum perfluorooctanoate** (**PFOA**) **and perfluorooctanesulfonic acid** (**PFOS**) **levels to colorectal cancer diagnosis in adults aged 21 and older**, **by year of diagnosis and period of residence in affected water district**

	**Resident since 1995 or before and including only CRC cases diagnosed 2000 or later (N=21,233 controls, 71 CRC cases)**				**Resident since 1990 or before and including only CRC cases diagnosed 2000 or later (N=15,533 controls, 60 CRC cases)**			
	**Cases**	**Controls**	Odds ratio (95% CI)*	**P**	**Cases**	**Controls**	Odds ratio (95% CI)*	**P**
						
***PFOS quartiles***								
First	42	5278	1.00		37	3884	1.00	
Second	12	5312	0.19 (0.09,0.38)	<0.00001	9	3884	0.18 (0.08,0.40)	0.00002
Third	7	5322	0.13 (0.06,0.27)	<0.00001	7	3864	0.14 (0.06,0.30)	<0.00001
Fourth	10	5321	0.12 (0.06,0.23)	<0.00001	7	3901	0.12 (0.06,0.24)	<0.00001
*Test for trend*				<0.00001				<0.00001
***PFOA quartiles***								
First	28	5284	1.00		25	3870	1.00	
Second	7	5325	0.25 (0.11,0.55)	0.001	9	3921	0.24 (0.10,0.56)	0.001
Third	21	5313	0.37 (0.19,0.70)	0.002	13	3828	0.32 (0.16,0.66)	0.002
Fourth	15	5311	0.43 (0.24,0.78)	0.005	13	3914	0.38 (0.20,0.72)	0.003
*Test for trend*				0.001				0.001

*Odds ratios adjusted for age, race, gender, socioeconomic status (years of education, annual household income, and employment status/disability), marital status, lifestyle factors, BMI, menopausal status, and comorbidity. Lifestyle factors include smoking (never, former, current); current alcohol consumption (yes/no); vegetarian diet (yes/no); exercise (regular exercise program (yes/no); comorbidity includes physician diagnosis of chronic comorbid condition (heart, kidney, liver, immune, connective tissue, and thyroid disease, stroke, hypertension, dyslipidemia, diabetes, chronic obstructive pulmonary disease, or asthma) and current treatment for hypertension or hyperlipidemia.

### Sensitivity analyses

**Figure 2:**
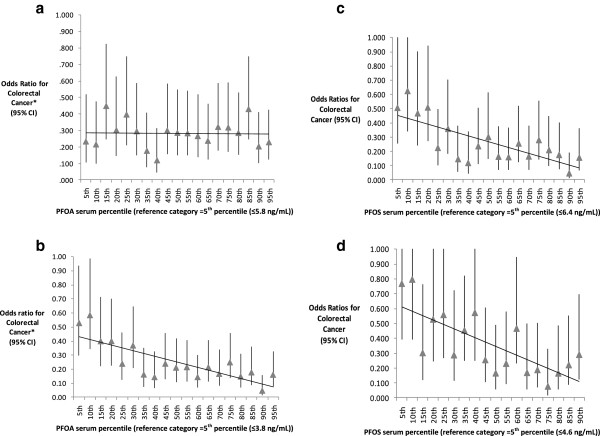
**Association of serum PFOA and PFOS levels to likelihood of colorectal cancer diagnosis. a**. All eligible participants (N=208 CRC cases, 47,151 controls). **b**. Sample restricted to those with PFOA serum values ≤ 20 ng/mL (N=84 CRC cases, 19,117 controls). **c**. All eligible participants (N=208 CRC cases, 47,151 controls). **d**. Sample restricted to those with PFOS serum values ≤ 20 ng/mL (N=118 CRC Cases, 23,287 Controls).

To determine whether the observed positive association of PFOA to CRC differed at exposure levels more typical of non-contaminated areas, we limited our subanalysis to adults with serum concentrations of PFOA ≤ 20 ng/mL (N = 19,201 adults, including 84 with CRC), with the lowest quartile (referent group) comprising blood levels similar to those in the U.S. general population [24]. Restricting the analysis to adults with these relatively low levels of exposure substantially strengthened the linear, inverse relationship of PFOA to CRC diagnosis, suggesting a possible threshold effect (age-adjusted OR for the highest vs. lowest quartile = 0.4 (CI 0.2, 0.7), p for trend =0.009). In this lower-exposure group, PFOA also showed a significant, linear association with CRC diagnosis when analyzed as a continuous variable (fully adjusted P = 0.001). Similarly, while the negative association of CRC to PFOA levels, in ventiles, did not appear linear in the overall population, a modest linear trend was evident among adults with PFOA levels ≤20 ng/mL, with likelihood of CRC diagnosis declining with increasing PFOA serum percentile relative to the lowest percentile range (5th percentile, ≤ 3.8 ng/mL) (Figure 2a, b). Using the contemporaneous U.S. population mean for PFOA (NHANES, 2003-4 [24]) as the referent category yielded similar results. In contrast, the probability of CRC diagnosis declined strongly with increasing PFOS serum percentile in both the overall population and in the restricted analyses (Figure 2c, d).

Removal of primary rectal cancer cases (N = 29) from the analysis did not alter the observed associations with either PFOA or PFOS. Similarly, removal of those who reported undergoing current treatment (N = 21) did not attenuate the findings (fully adjusted OR’s for the highest vs. the lowest quartile for PFOS and PFOA, respectively = 0.2 (CI 0.1, 0.3), p for trend < 0.00001; and 0.6 (0.4, 0.95), p for trend < 0.001), nor did removal of those who had received chemotherapy (N = 109). Main analyses were also repeated in all participants reporting a physician diagnosis of primary colorectal cancer (N = 281); inclusion of these additional unconfirmed cases did not appreciably change either the strength or the magnitude of the observed associations (fully adjusted OR’s for the highest vs. the lowest quartile for PFOS and PFOA, respectively = 0.2 (CI 0.2, 0.3), p for trend < 0.00001; and 0.6 (0.4, 0.9), p for trend < 0.01). In contrast, another gut-absorbed compound, folate, showed no relation to CRC diagnosis (p = 0.68), and age-adjusted serum folate levels did not differ by disease status (p > 0.4). Associations of CRC to the 5 additional PFCs were inconsistent, with 2 (C6, C10) showing significant positive associations (p < 0.001), 2 (C6s, C9) showing significant inverse associations (p ≤ 0.01), and one (C7) showing no association (p = 0.7) with CRC.

## Discussion

To our knowledge, this is the first large, community-based study to examine the potential links between CRC and environmental PFAAs, and the first to examine in detail the association of CRC to PFOA and PFOS. Our findings indicate a significant inverse association between both PFOS and PFOA and diagnosis of CRC in this large cross-sectional study. This association between PFOS and CRC was particularly pronounced, with a strong linear inverse relationship that remained robust even at lower exposure levels. After adjustment for multiple potential confounders, those with serum PFOS levels in the highest quartile were approximately 80% less likely to have received a diagnosis of CRC; likewise, those in the highest quartile of PFOA were approximately 40% less likely to have been diagnosed with CRC. These inverse associations were stronger with CRC diagnosed within the previous 6 years and in long term residents of the affected water districts. The relationship between PFOA and CRC was also more pronounced in men and leaner adults, and showed a strong linear trend only at lower serum concentrations comparable to those observed in the general US general population, suggesting a possible threshold or saturation effect for this PFAA.

Experimental studies have indicated carcinogenic effects of both PFOA and PFOS in rodent models, at least at high exposures; however, no associations between these PFAA’s and CRC have been reported. Moreover, published studies regarding potential links between PFAAs and cancer in humans are sparse and have yielded inconclusive findings. Two European studies, including a large Danish nested cohort study (N = 1240 liver, bladder, pancreatic, and prostate cancer patients) [26] and a small Greek case–control study (N = 40 cancer patients) [27], reported no associations between serum levels of either PFAA and cancer diagnosis. While two previous investigations in a cohort of PFOS-exposed workers suggested a possible positive relation between high PFOS exposure and CRC, the findings were non-significant, based on very small case numbers, and reliant on indirect measures of exposure [14,15].

Consistent with previous studies, we found the likelihood of CRC diagnosis to increase strongly with age [2]. Menopause was also positively associated with CRC diagnosis, in agreement with findings of some [28-31] but not other investigations [32-34]. Likewise, those on lipid-lowering medications were also less likely to have been diagnosed with CRC, similar to findings from some [35-37], but not all studies [38-41]. In this study, those with comorbid conditions were more likely to have received a diagnosis of CRC. Previous studies have shown comorbidity to predict earlier stage of CRC diagnosis [42] as well as reduced survival in CRC patients [43]. Several but not all [44] studies have reported increased CRC risk in association with specific conditions, including hypertension, [45,46] diabetes [45-47], and other chronic disorders [45,46], although research specifically assessing the association between overall comorbidity and diagnosed CRC is sparse. BMI was unrelated to CRC diagnosis overall in the current study after adjustment for other sociodemographic and medical factors. However, stratification by gender revealed BMI to be significantly associated with CRC in men but not in women (adjusted ORs for BMI ≥ 25 in men vs. women, respectively = 1.9 (CI 1.1, 3.2) vs. 1.0 (0.6, 1.5), p for interaction = 0.01), findings consistent with those of previous investigations [48].

The strengths of the study include the population-based design, the large sample size, and the high study participation rates in an Appalachian region. Additional strengths include our ability to evaluate persistent biomarkers of PFAA exposure obtained concurrently with survey and medical record information regarding CRC and other chronic conditions, the measurement of a broad array of biomarkers, and the extensive information available on potential confounders and effect modifiers. Ascertainment of CRC was based on participant-reported physician diagnosis and confirmed by chart review. We were also able to assess the association of CRC to PFOA and PFOS over a wide range of serum concentrations, helping to clarify the possible modifying effects of exposure level.

Our study has several important limitations as well. While human half-lives of PFOA and PFOS are long (up to 8.5 [49] and 5.4 years [50], respectively), random misclassification error due to the use of current PFC levels as proxy measures of etiologically relevant exposures may have biased our results toward the null. Most importantly, the cross-sectional nature of the data limits our ability to determine direction of causality. However, the persistence of PFOA and PFOS in human tissues, coupled with the strong inverse associations observed with recent CRC diagnosis in long-term residents, and the robust inverse association of both PFOA and especially, PFOS to CRC at varying exposure levels suggest that a causal relationship may be plausible. While it remains possible that differences in absorption due to the disease or treatment could help explain the observed effects, several lines of reasoning suggest this may not be the case. Adjustment for anemia and for GI symptoms potentially related to reduced absorption did not alter the observed associations, nor did removal of those currently on treatment or who had received chemotherapy. Another substance absorbed primarily through the ileum, folate, showed no relation to CRC diagnosis, and age-adjusted serum folate levels did not differ by disease status. Moreover, the associations of prevalent CRC to serum levels of other PFCs were inconsistent, with 3 of the 5 appearing unrelated or positively related to CRC diagnosis. In addition, CRC has been characterized by reduced gut motility and over-efficient enterohepatic circulation [51], which would be expected to lead to increased, rather than reduced PFAA serum levels [52-54]. Nonetheless, while these observations collectively suggest that altered PFAA absorption due to CRC and CRC treatment is unlikely to explain the robust inverse associations observed in this study, reverse causality remains a possibility and caution is thus warranted in interpreting our findings.

Additional limitations include lack of information on CRC stage at diagnosis and on certain risk factors for CRC, including inherited genetic alterations, history of inflammatory bowel disease, and specific dietary factors. As only self-reported physician diagnoses of CRC and other cancers were confirmed by chart review, unascertainment due to under-reporting remains possible. However, such underascertainment would be expected to bias the observed associations toward the null, and thus is unlikely to explain our findings. Exclusion of eligible participants with missing data on covariates may have introduced selection bias. Fortunately, the percentage with missing data was small (3.8%). Moreover, given those with missing data did not differ in either their PFC levels or in the incidence of CRC, their exclusion would not be expected to alter the observed associations. Diagnosis could not be validated for a substantial portion (25.8%) of those reporting a physician diagnosis of CRC. However, inclusion of both validated and non-validated cases in the analysis did not alter our findings. Diagnosis was also verified for 100% of participants who reported primary CRC and for whom information was available, indicating excellent reliability and validity of self report diagnosis in this study sample.

Although considerable efforts were made to contact former residents of affected counties, some otherwise eligible adults who moved out of the area may not have participated in the study, possibly introducing sampling bias. Our study population did not include those who had died from CRC and thus may not be representative of adults with more aggressive or advanced disease, limiting generalizability. However, the relation of both PFAAs to CRC diagnosis was stronger in those diagnosed more recently, and remained robust even in those diagnosed within a year of study health assessment, suggesting that survival bias is unlikely to account for our findings. Unmeasured confounding might also help explain our findings, although our ability to control for a large number of both known and potential risk factors for CRC renders this possibility less probable. Finally, some degree of method uncertainty in PFC detection is also possible. However, such uncertainty would be expected to attenuate observed associations and is thus unlikely to explain our findings. In addition, that the laboratory performing the assays followed U.S. Food and Drug Administration (FDA) approved procedures, and assays met the FDA standard for assay precision suggest that significant method uncertainty is unlikely.

If the association with either PFAA proves causal, possible physiologic explanations may include immune modulation, specific anti-cancer effects, including anti-proliferative, apoptotic and differentiating effects, and physiological changes related to the chemical properties of these PFAAs. While animal studies have yielded somewhat inconsistent results [17,55], a number of investigations in both rodent models and human cell lines suggest PFOA and PFOS can have potent anti-inflammatory effects. For example, in mouse models of inflammation, oral administration of PFOS in doses comparable to those reported in exposed human populations significantly reduced serum TNF-α and decreased peritoneal lavage fluid TNF-α and interleukin-6 levels in response to an immune challenge [17]. Similarly, relatively low doses of PFOA were found to dramatically reduce multiple signs of peripheral inflammation in rats, an effect that did not appear mediated by glucocorticoid receptors [56]. Consistent with these findings, recent in vitro studies of human leukocytes showed PFOS to decrease release of TNF-α following lipopolysaccharide stimulation [57].

The observed anti-inflammatory effects of these PFAAs may be mediated in part by activation of peroxisome proliferator-activated receptors (PPARs). PPARs are transcription factors that belong to the superfamily of nuclear receptors whose natural activating ligands are lipid-derived substrates. A growing body of experimental evidence suggests PFOA and PFOS are potent PPAR ligands, acting as agonists for both the alpha (PPAR-α) and gamma isotypes (PPAR-γ) [17,18,58-60]. PPARs are expressed on macrophages [61], and are highly expressed in intestinal [59] and other tissues [18]. These receptors are now thought to play a major role in the regulation of inflammatory responses [11,18,62-64]. For example, PPAR-γ has been shown to down-regulate the expression of inflammatory cytokines, inhibit major inflammatory signaling pathways [65], and direct immune cell differentiation towards anti-inflammatory phenotypes [66].

PPARs are also thought to play a key role in the differentiation, apoptosis and proliferation of colon and other tumor cells [10,11,63], contributing to the growing research interest in these receptors and their ligands as potential chemoprevention and chemotherapeutic agents [10,11]. Studies in rodent models of colorectal and other tumors, as well as in human normal and tumor cell lines have shown PPAR-*γ* ligands to exert powerful anti-cancer effects; these effects appear mediated by the differentiating, pro-apoptotic, and anti-proliferative effects on malignant cells, as well as by the anti-inflammatory actions of PPAR-γ [10,11]. Likewise, PPAR-α has also been implicated in the inception and progression of CRC. For example, PPAR-α ligands induce apoptosis in human tumor cell lines and PPAR-*α* expression is reduced in human tubular adenomas and colonic adenocarinoma cells compared to normal human colonic mucosa [63,67]. In addition, both PPAR-*α* and PPAR-*γ* ligands have been shown to suppress polyp formation in an animal model of familial polyposis [67,68].

Finally, PFOS and PFOA are also surfactants, characterized by strong hydrophobic properties [69], which could, in turn, contribute to a protective effect against CRC. While this possibility remains unexplored in PFAAs, other compounds with hydrophobic properties have been shown to induce cell cycle arrest and apoptosis of human colon cancer [70] and other tumor cells [71].

## Conclusions

In this cross-sectional study of a large Appalachian population, we found a strong inverse linear association between PFOS and likelihood of CRC diagnosis and a significant, although more modest inverse association between PFOA and diagnosis of CRC. If confirmed in prospective investigations, these findings could aid in identifying new strategies for CRC prevention and treatment and help inform future studies regarding possible mechanisms underlying the pathogenesis of this of this common and often fatal cancer.

## Competing interest

None of the authors have actual or potential competing financial or non-financial interests. The authors of this manuscript declare that their ability to design, conduct, interpret, or publish research was unimpeded by and fully independent of the court and/or settling parties.

## Authors’ contributions

KEI conceived and designed the study, helped prepare the data and conducted the analyses, interpreted the findings, and drafted the paper; AMD contributed to data acquisition and interpretation of findings, and helped to draft the manuscript; JW contributed to interpretation of the data and to critical revision of the manuscript; SF participated in acquiring and preparing the data, and in revising the paper. All authors read and approved the final manuscript.

## Pre-publication history

The pre-publication history for this paper can be accessed here:

http://www.biomedcentral.com/1471-2407/14/45/prepub

## References

[B1] U.S. Cancer Statistics Working Group, United States Cancer Statistics 1999–2006 Incidence and Mortality Web-based Report 2010 Atlanta (GA): Department of Health and Human Services, Centers for Disease Control and Prevention, and National Cancer Institute

[B2] Edwards B Ward E Kohler B Annual report to the nation on the status of cancer, 1975–2006, featuring colorectal cancer trends and impact of interventions (risk factors, screening, and treatment) to reduce future rates Cancer Res 2010 116 3 544 573 10.1002/cncr.24760 PMC3619726 19998273

[B3] Hao YP Jemal A Zhang XY Ward EM Trends in colorectal cancer incidence rates by age, race/ethnicity, and indices of access to medical care, 1995–2004 (United States) Cancer Cause Control 2009 20 10 1855 1863 10.1007/s10552-009-9379-y 19543799

[B4] Kojima M Wakai K Tokudome S Bowel movement frequency and risk of colorectal cancer in a large cohort study of Japanese men and women Br J Cancer 2004 90 7 1397 1401 10.1038/sj.bjc.6601735 15054462 PMC2409677

[B5] Watanabe T Nakaya N Kurashima K Kuriyama S Tsubono Y Tsuji I Constipation, laxative use and risk of colorectal cancer: The Miyagi Cohort Study Eur J Cancer 2004 40 14 2109 2115 10.1016/j.ejca.2004.06.014 15341986

[B6] Cuzick J Otto F Baron JA Aspirin and non-steroidal anti-inflammatory drugs for cancer prevention: an international consensus statement Lancet Oncol 2009 10 5 501 507 10.1016/S1470-2045(09)70035-X 19410194

[B7] Thun MJ Henley SJ Patrono C Nonsteroidal Anti-inflammatory Drugs as Anticancer Agents: Mechanistic, Pharmacologic, and Clinical Issues J Natl Cancer Inst 2002 94 No. 4 252 266 10.1093/jnci/94.4.252 11854387

[B8] Jalving M Koornstra JJ De Jong S De Vries EG Kleibeuker JH Review article: the potential of combinational regimen with non-steroidal anti-inflammatory drugs in the chemoprevention of colorectal cancer Aliment Pharmacol Ther 2005 21 4 321 339 10.1111/j.1365-2036.2005.02335.x 15709983

[B9] Harris RE Beebe-Donk J Doss H Burr DD Aspirin, ibuprofen, and other non-steroidal anti-inflammatory drugs in cancer prevention: a critical review of non-selective COX-2 blockade (review) Oncol Rep 2005 13 4 559 583 15756426

[B10] Hatton JL Yee LD Clinical Use of PPARγ Ligands in Cancer. PPAR Research 2008 doi:10.1155/2008/159415:1–13 10.1155/2008/159415 PMC2605846 19125177

[B11] Peters JM Shah YM Gonzalez FJ The role of peroxisome proliferator-activated receptors in carcinogenesis and chemoprevention Nat Rev Cancer 2012 12 181 195 22318237 10.1038/nrc3214 PMC3322353

[B12] Weyer PJ Cerhan JR Kross BC Municipal drinking water nitrate level and cancer risk in older women: The Iowa Women's Health Study Epidemiology 2001 12 3 327 338 10.1097/00001648-200105000-00013 11338313

[B13] Doyle TJ Zheng W Cerhan JR The association of drinking water source and chlorination by-products with cancer incidence among postmenopausal women in Iowa: A prospective cohort study Am J Public Health 1997 87 7 1168 1176 10.2105/AJPH.87.7.1168 9240108 PMC1380892

[B14] Grice MM Alexander BH Hoffbeck R Kampa DM Self-reported medical conditions in perfluorooctanesulfonyl fluoride manufacturing workers J Occup Environ Med 2007 49 7 722 729 10.1097/JOM.0b013e3180582043 17622844

[B15] Olsen GW Burlew MM Marshall JC Burris JM Mandel JH Analysis of episodes of care in a perfluorooctanesulfonyl fluoride production facility J Occup Environ Med 2004 46 8 837 846 10.1097/01.jom.0000135546.70469.87 15300136

[B16] Corsini E Sangiovanni E Avogadro A In vitro characterization of the immunotoxic potential of several perfluorinated compounds (PFCs) Toxicol Appl Pharm 2012 258 2 248 255 10.1016/j.taap.2011.11.004 22119708

[B17] Mollenhauer MA Bradshaw SG Fair PA McGuinn WD Peden-Adams MM Effects of perfluorooctane sulfonate (PFOS) exposure on markers of inflammation in female B6C3F1 mice J Environ Sci Health A Tox Hazard Subst Environ Eng 2011 46 2 97 108 10.1080/10934529.2011.532418 21170772

[B18] DeWitt JC Shnyra A Badr MZ Immunotoxicity of Perfluorooctanoic Acid and Perfluorooctane Sulfonate and the Role of Peroxisome Proliferator-Activated Receptor Alpha Crit Rev Toxicol 2009 39 1 76 94 10.1080/10408440802209804 18802816

[B19] Steenland K Tinker S Shankar A Ducatman A Association of Perfluorooctanoic Acid (PFOA) and Perfluorooctane Sulfonate (PFOS) with Uric Acid among Adults with Elevated Community Exposure to PFOA Environ Health Perspect 2010 118 229 233 20123605 10.1289/ehp.0900940 PMC2831922

[B20] Frisbee SJ Brooks AP Jr Maher A The C8 Health Project: Design, Methods, and Participants Environ Health Perspect 2009 117 12 1873 1882 10.1289/ehp.0800379 20049206 PMC2799461

[B21] Bartell S Calafat A Lyu C Kato K Ryan P Steenland K Rate of decline in serum PFOA concentrations after granular activated carbon filtration at two public water systems in Ohio and West Virginia Environ Health Perspect 2010 118 2 222 228 20123620 10.1289/ehp.0901252 PMC2831921

[B22] Steenland K Tinker S Frisbee S Ducatman A Vaccarino V Association of perfluorooctanoic acid and perfluorooctane sulfonate with serum lipids among adults living near a chemical plant Am J Epidemiol 2009 170 10 1268 1278 10.1093/aje/kwp279 19846564

[B23] FDA U Guidance for industry: bioanalytical method validation 2001 Rockville, MD: CDER, ed.

[B24] Calafat AM Wong L-Y Kuklenyik Z Reidy JA Needham LL Polyfluoroalkyl Chemicals in the U.S. Population: Data from the National Health and Nutrition Examination Survey (NHANES) 2003–2004 and Comparisons with NHANES 1999–2000 Environ Health Perspect 2007 115 11 1596 1602 10.1289/ehp.10598 18007991 PMC2072821

[B25] Nelson JW Hatch EE Webster TF Exposure to polyfluoroalkyl chemicals and cholesterol, body weight, and insulin resistance in the general U.S. population Environ Health Perspect 2010 118 2 197 202 20123614 10.1289/ehp.0901165 PMC2831917

[B26] Eriksen KT Sorensen M McLaughlin JK Perfluorooctanoate and Perfluorooctanesulfonate Plasma Levels and Risk of Cancer in the General Danish Population J Natl Cancer Inst 2009 101 8 605 609 10.1093/jnci/djp041 19351918

[B27] Vassiliadou I Costopoulou D Ferderigou A Leondiadis L Levels of perfluorooctanesulfonate (PFOS) and perfluorooctanoate (PFOA) in blood samples from different groups of adults living in Greece Chemosphere 2010 80 10 1199 1206 10.1016/j.chemosphere.2010.06.014 20619872

[B28] Franceschi S Gallus S Talamini R Tavani A Negri E La Vecchia C Menopause and colorectal cancer Br J Cancer 2000 82 11 1860 1862 10.1054/bjoc.1999.1084 10839302 PMC2363225

[B29] Peters RK Pike MC Chang WW Mack TM Reproductive factors and colon cancers Br J Cancer 1990 61 5 741 748 10.1038/bjc.1990.166 2337511 PMC1971601

[B30] Wu AH Paganini-Hill A Ross RK Henderson BE Alcohol, physical activity and other risk factors for colorectal cancer: a prospective study Br J Cancer 1987 55 6 687 694 10.1038/bjc.1987.140 3620314 PMC2002031

[B31] van Wayenburg CA van der Schouw YT van Noord PA Peeters PH Age at menopause, body mass index, and the risk of colorectal cancer mortality in the Dutch Diagnostisch Onderzoek Mammacarcinoom (DOM) cohort Epidemiology 2000 11 3 304 308 10.1097/00001648-200005000-00013 10784248

[B32] Wu-Williams AH Lee M Whittemore AS Reproductive factors and colorectal cancer risk among Chinese females Cancer Res 1991 51 9 2307 2311 2015594

[B33] Martinez ME Grodstein F Giovannucci E A prospective study of reproductive factors, oral contraceptive use, and risk of colorectal cancer Cancer Epidem Biomar 1997 6 1 1 5 8993789

[B34] Kampman E Potter JD Slattery ML Caan BJ Edwards S Hormone replacement therapy, reproductive history, and colon cancer: a multicenter, case–control study in the United States Cancer Causes Control 1997 8 2 146 158 10.1023/A:1018459911147 9134238

[B35] Simon MS Rosenberg CA Rodabough RJ Prospective analysis of association between use of statins or other lipid-lowering agents and colorectal cancer risk Ann Epidemiol 2012 22 1 17 27 10.1016/j.annepidem.2011.10.006 22056480 PMC3804112

[B36] Poynter JN Gruber SB Higgins PD Statins and the risk of colorectal cancer N Engl J Med 2005 352 21 2184 2192 10.1056/NEJMoa043792 15917383

[B37] Bonovas S Filioussi K Flordellis CS Sitaras NM Statins and the risk of colorectal cancer: a meta-analysis of 18 studies involving more than 1.5 million patients J Clin Oncol 2007 25 23 3462 3468 10.1200/JCO.2007.10.8936 17687150

[B38] Jacobs EJ Rodriguez C Brady KA Connell CJ Thun MJ Calle EE Cholesterol-lowering drugs and colorectal cancer incidence in a large United States cohort J Natl Cancer Inst 2006 98 1 69 72 10.1093/jnci/djj006 16391373

[B39] Vinogradova Y Hippisley-Cox J Coupland C Logan RF Risk of colorectal cancer in patients prescribed statins, nonsteroidal anti-inflammatory drugs, and cyclooxygenase-2 inhibitors: nested case–control study Gastroenterology 2007 133 2 393 402 10.1053/j.gastro.2007.05.023 17681160

[B40] Setoguchi S Glynn RJ Avorn J Mogun H Schneeweiss S Statins and the risk of lung, breast, and colorectal cancer in the elderly Circulation 2007 115 1 27 33 17179016 10.1161/CIRCULATIONAHA.106.650176

[B41] Singh H Mahmud SM Turner D Xue L Demers AA Bernstein CN Long-term use of statins and risk of colorectal cancer: a population-based study Am J Gastroenterol 2009 104 12 3015 3023 10.1038/ajg.2009.574 19809413

[B42] Zafar SY Abernethy AP Abbott DH Comorbidity, age, race and stage at diagnosis in colorectal cancer: a retrospective, parallel analysis of two health systems Bmc Cancer 2008 8 345 1 9 doi:10.1186/1471-2407-8-345 19032772 10.1186/1471-2407-8-345 PMC2613913

[B43] Yancik R Wesley MN Ries LAG Comorbidity and age as predictors of risk for early mortality of male and female colon carcinoma patients - A population-based study Cancer 1998 82 11 2123 2134 10.1002/(SICI)1097-0142(19980601)82:11<2123::AID-CNCR6>3.0.CO;2-W 9610691

[B44] Kune GA Kune S Watson LF Colorectal cancer risk, chronic illnesses, operations and medications: case control results from the Melbourne Colorectal Cancer Study. 1988 Int J Epidemiol 2007 36 5 951 957 10.1093/ije/dym193 17921195

[B45] Stocks T Lukanova A Johansson M Components of the metabolic syndrome and colorectal cancer risk; a prospective study Int J Obes (Lond) 2008 32 2 304 314 10.1038/sj.ijo.0803713 17878894

[B46] Giovannucci E Metabolic syndrome, hyperinsulinemia, and colon cancer: a review Am J Clin Nutr 2007 86 3 s836 842 18265477 10.1093/ajcn/86.3.836S

[B47] Larsson SC Orsini N Wolk A Diabetes mellitus and risk of colorectal cancer: a meta-analysis J Natl Cancer Inst 2005 97 22 1679 1687 10.1093/jnci/dji375 16288121

[B48] Pischon T Lahmann PH Boeing H Body size and risk of colon and rectal cancer in the European prospective investigation into cancer and nutrition (EPIC) J Natl Cancer Inst 2006 98 13 920 931 10.1093/jnci/djj246 16818856

[B49] Seals R Bartell SM Steenland K Accumulation and clearance of perfluorooctanoic acid (PFOA) in current and former residents of an exposed community Environ Health Perspect 2011 119 1 119 124 20870569 10.1289/ehp.1002346 PMC3018490

[B50] Olsen GW Burris JM Ehresman DJ Half-life serum elimination of perfluorooctanesulfonate, perfluorohexanesulfonate, and perfluorooctanoate in retired fluorochemical production workers Environ Health Perspect 2007 115 9 1298 1305 10.1289/ehp.10009 17805419 PMC1964923

[B51] Lewis SJ Heaton KW The metabolic consequences of slow colonic transit Am J Gastroenterol 1999 94 8 2010 2016 10.1111/j.1572-0241.1999.01271.x 10445521

[B52] Kudo N Kawashima Y Toxicity and toxicokinetics of perfluorooctanoic acid in humans and animals J Toxicol Sci 2003 28 2 49 57 10.2131/jts.28.49 12820537

[B53] Jandacek RJ Tso P Enterohepatic circulation of organochlorine compounds: a site for nutritional intervention J Nutr Biochem 2007 18 3 163 167 10.1016/j.jnutbio.2006.12.001 17296488

[B54] Lau C Anitole K Hodes C Lai D Pfahles-Hutchens A Seed J Perfluoroalkyl acids: a review of monitoring and toxicological findings Toxicol Sci 2007 99 2 366 394 10.1093/toxsci/kfm128 17519394

[B55] Dong GH Zhang YH Zheng L Liang ZF Jin YH He QC Subchronic effects of perfluorooctanesulfonate exposure on inflammation in adult male C57BL/6 mice Environ Toxicol 2012 27 5 285 296 10.1002/tox.20642 20737580

[B56] Taylor BK Kriedt C Nagalingam S Dadia N Badr M Central administration of perfluorooctanoic acid inhibits cutaneous inflammation Inflamm Res 2005 54 6 235 242 10.1007/s00011-005-1350-0 15973506

[B57] Brieger A Bienefeld N Hasan R Goerlich R Haase H Impact of perfluorooctanesulfonate and perfluorooctanoic acid on human peripheral leukocytes Toxicol In Vitro 2011 25 4 960 968 10.1016/j.tiv.2011.03.005 21397682

[B58] Vanden Heuvel J Thompson J Frame S Gillies P Differential activation of nuclear receptors by perfluorinated fatty acid analogs and natural fatty acids: a comparison of human, mouse, and rat peroxisome proliferator-activated receptor-alpha, -beta, and -gamma, liver X receptor-beta, and retinoid X receptor-alpha Toxicol Sci 2006 92 2 476 489 10.1093/toxsci/kfl014 16731579

[B59] Mohapatra SK Guri AJ Climent M Immunoregulatory Actions of Epithelial Cell PPAR γ at the Colonic Mucosa of Mice with Experimental Inflammatory Bowel Disease PLoS ONE 2010 5 4 e10215 10.1371/journal.pone.0010215 20422041 PMC2857885

[B60] Nakagawa T Ramdhan DH Tanaka N Modulation of ammonium perfluorooctanoate-induced hepatic damage by genetically different PPARalpha in mice Arch Toxicol 2012 86 1 63 74 10.1007/s00204-011-0704-3 21499893 PMC6594146

[B61] Giaginis C Tsantili-Kakoulidou A Theocharis S Peroxisome proliferator-activated receptors (PPARs) in the control of bone metabolism Fundam Clin Pharmacol 2007 21 3 231 244 10.1111/j.1472-8206.2007.00486.x 17521292

[B62] Lefebvre P Chinetti G Fruchart JC Staels B Sorting out the roles of PPAR alpha in energy metabolism and vascular homeostasis J Clin Invest 2006 116 3 571 580 10.1172/JCI27989 16511589 PMC1386122

[B63] Martinasso G Oraldi M Trombetta A Involvement of PPARs in Cell Proliferation and Apoptosis in Human Colon Cancer Specimens and in Normal and Cancer Cell Lines PPAR Res 2007 2007 93416 17389773 10.1155/2007/93416 PMC1852897

[B64] Clark RB The role of PPARs in inflammation and immunity J Leukoc Biol 2002 71 3 388 400 11867676

[B65] Giaginis C Giagini A Theocharis S Peroxisome proliferator-activated receptor-y (PPAR-y) ligands as potential therapeutic agents to treat arthritis Pharmacol Res 2009 60 160 169 10.1016/j.phrs.2009.02.005 19646655

[B66] Martin H Role of PPAR-gamma in inflammation. Prospects for therapeutic intervention by food components Mutat Res 2010 690 1–2 57 63 20973164 10.1016/j.mrfmmm.2009.09.009

[B67] Jackson L Wahli W Michalik L Potential role for peroxisome proliferator activated receptor (PPAR) in preventing colon cancer Gut 2003 52 9 1317 1322 10.1136/gut.52.9.1317 12912864 PMC1773786

[B68] Mutoh M Niho N Wakabayashi K Concomitant suppression of hyperlipidemia and intestinal polyp formation by increasing lipoprotein lipase activity in Apc-deficient mice Biol Chem 2006 387 4 381 385 16606335 10.1515/BC.2006.051

[B69] Volkel W Genzel-Boroviczeny O Demmelmair H Perfluorooctane sulphonate (PFOS) and perfluorooctanoic acid (PFOA) in human breast milk: results of a pilot study Int J Hyg Environ Health 2008 211 3–4 440 446 17870667 10.1016/j.ijheh.2007.07.024

[B70] Komizu Y Ueoka H Goto K Ueoka R Remarkable inhibitory effects of hybrid liposomes on growth of human colon cancer cells through induction of cell cycle arrest along with apoptosis Int J Nanomedicine 2011 6 1913 1920 21931486 10.2147/IJN.S24160 PMC3173053

[B71] Fayad W Rickardson L Haglund C Identification of Agents that Induce Apoptosis of Multicellular Tumour Spheroids: Enrichment for Mitotic Inhibitors with Hydrophobic Properties Chem Biol Drug Des 2011 78 4 547 557 10.1111/j.1747-0285.2011.01170.x 21726416

